# Systematic Screening of Associations between Medication Use and Risk of Neurodegenerative Diseases Using a Mendelian Randomization Approach

**DOI:** 10.3390/biomedicines11071930

**Published:** 2023-07-07

**Authors:** Wenjing Wang, Linjing Zhang, Wen Cao, Kailin Xia, Junyan Huo, Tao Huang, Dongsheng Fan

**Affiliations:** 1 Department of Neurology, Peking University Third Hospital, Beijing 100191, China; 2 Beijing Key Laboratory of Biomarker and Translational Research in Neurodegenerative Diseases, Beijing 100191, China; 3 Key Laboratory for Neuroscience, National Health Commission/Ministry of Education, Peking University, Beijing 100191, China; 4 Department of Epidemiology and Biostatistics, School of Public Health, Peking University, Beijing 100191, China; 5 Key Laboratory of Molecular Cardiovascular Sciences, Peking University, Ministry of Education, Beijing 100191, China

**Keywords:** neurodegenerative diseases, Alzheimer’s disease (AD), Parkinson’s disease (PD), amyotrophic lateral sclerosis (ALS), medication use

## Abstract

Background: Systematically assessing the causal associations between medications and neurodegenerative diseases is significant in identifying disease etiology and novel therapies. Here, we investigated the putative causal associations between 23 existing medication categories and major neurodegenerative diseases (NDs), including Alzheimer’s disease (AD), Parkinson’s disease (PD), and amyotrophic lateral sclerosis (ALS). Methods: A two-sample mendelian randomization (MR) approach was conducted. Estimates were calculated using the inverse-variance weighted (IVW) method as the main model. A sensitivity analysis and a pleiotropy analysis were performed to identify potential violations. Results: Genetically predisposition to antihypertensives (OR = 0.809, 95% CI = 0.668–0.981, *p* = 0.031), thyroid preparations (OR = 0.948, 95% CI = 0.909–0.988, *p* = 0.011), and immunosuppressants (OR = 0.879, 95% CI = 0.789–0.979, *p* = 0.018) was associated with a decreased risk of AD. Genetic proxies for thyroid preparations (OR = 0.934, 95% CI = 0.884–0.988, *p* = 0.017), immunosuppressants (OR = 0.825, 95% CI = 0.699–0.973, *p* = 0.022), and glucocorticoids (OR = 0.862, 95% CI = 0.756–0.983, *p* = 0.027) were causally associated with a decreased risk of PD. Genetically determined antithrombotic agents (OR = 1.234, 95% CI = 1.042–1.461, *p* = 0.015), HMG CoA reductase inhibitors (OR = 1.085, 95% CI = 1.025–1.148, *p* = 0.005), and salicylic acid and derivatives (OR = 1.294, 95% CI = 1.078–1.553, *p* = 0.006) were associated with an increased risk of ALS. Conclusions: We presented a systematic view concerning the causal associations between medications and NDs, which will promote the etiology discovery, drug repositioning and patient management for NDs.

## 1. Introduction

Neurodegenerative diseases (NDs), represented by Alzheimer’s disease (AD), Parkinson’s disease (PD), and amyotrophic lateral sclerosis (ALS), are characterized by a progressive loss of selectively vulnerable populations of neurons [1]. The World Health Organization (WHO) predicts that NDs will overtake cancer and become the second-most prevalent cause of death after cardiovascular diseases in the next 20 years. However, the etiology of NDs remains largely unknown. The current treatments available only manage the symptoms or halt the progression of the disease [2]. Therefore, there is an urgent and unmet need for the identification of disease mechanisms and new clinical treatments for NDs.

Identifying the causal associations between existing medications and NDs is convenient and meaningful to probe disease etiology and identify novel therapies [3,4]. Existing medications target a range of mechanisms, including immune and inflammatory responses [5], lipid metabolism [6], oxidative stress [7], platelet function [8], and others, all of which are involved in either neuron survival or degeneration. It was famously stated by the pharmacologist and Nobel laureate James Black that “the most fruitful basis for the discovery of a new drug is to start with an old drug” [9]. Drug repurposing is substantially more cost-effective than designing and optimizing a new drug, since the safety and tolerability have already been established in clinical practice [3]. However, it is still quite challenging to systematically analyze the causal effects of thousands of existing clinical drugs on NDs. Randomized controlled trials (RCTs) or observational studies need heavy investments in people and money. In addition, RCTs are sometimes unethical, and observational studies often suffer from confounding or reverse causation [10,11]. Therefore, in these traditional studies, it is almost impossible to achieve reliable and high-throughput screening for the causal associations between existing medications and NDs.

During the last decade, significant advances in large-scale genome-wide association studies (GWASs) and the powerful statistical tool mendelian randomization (MR) have provided us with the chance to systematically and cost-effectively assess the causal relationship between different phenotypes. GWASs have identified thousands of single nucleotide polymorphisms (SNPs) associated with major diseases and phenotypes [12]. These SNPs can be employed as unconfounded proxies (instrumental variables) for exposures by MR to estimate the causal effect of the exposure on the outcome of interest [13]. MR analysis relies on three assumptions regarding the genetic variant used as an instrumental variable (IV). Firstly, the IV is related to the exposure being investigated. Secondly, the IV is not influenced by confounding variables. Thirdly, the effect of the IV on the outcome is through the exposure of interest [13,14]. In this framework, MR minimizes the biased results arising from confounding or reverse causation in observational studies and has analogies with RCTs because the two alleles of an SNP are randomly segregated in gamete formation [13]. Moreover, MR only uses publicly available GWAS data, providing an obvious advantage over traditional clinical studies in terms of high throughput.

Therefore, the objective of this study is to provide a comprehensive view of the potential causal associations between existing medications and major neurodegenerative diseases (AD, PD and ALS) using these systematic medication-use GWAS data and the MR approach. GWAS data for 23 medication categories based on the UK Biobank have been obtained, and some have already been used in MR studies to evaluate the effects of opioid use on major depression [15] and cardiovascular diseases [16], as well as the effects of immunosuppressants on Parkinson’s disease [17]. Firstly, we will systematically screen the associations between 23 medication categories and neurodegenerative diseases (NDs) using medication-use GWAS data. Secondly, we will evaluate the causal link between the primary diseases that these medications are used for and NDs, before and after removing instrumental variants associated with medications, in order to clarify the medication-ND relationship. This study will identify potential disease mechanisms and new treatment opportunities, while also avoiding side effects in the treatment of neurodegenerative diseases.

## 2. Materials and Methods

Two-sample MR was performed to systematically explore the causal associations between 23 medication categories and three major neurodegenerative diseases (AD, PD and ALS). The two-sample MR approach is a frequently used method in MR studies, where the exposure and outcome data are derived from separate datasets, usually based on summary statistics offered by GWAS consortia [14,18].

### 2.1. Exposure Data: GWAS Summary Statistics for 23 Medication Categories

**Figure 1:**
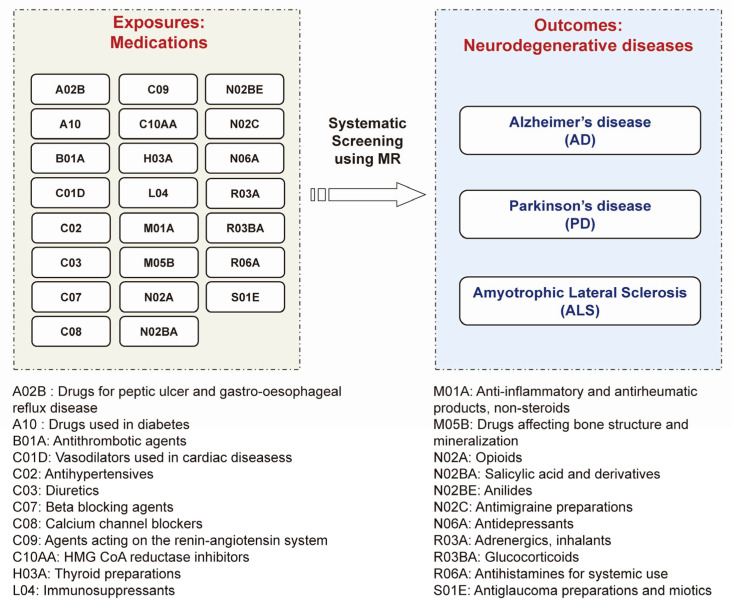
The concept and design of this study. Using MR approach to systematically screen the causal pharmacological effects of 23 categories of medications (exposures) and 3 major neurodegenerative diseases (outcomes) including Alzheimer’s disease (AD), Parkinson’s disease (PD), and amyotrophic lateral sclerosis (ALS).

For medication-taking traits, the GWAS summary statistics were obtained from Wu et al.’s study [12]. Briefly, Wu et al. employed self-reported regular medication data (no medication duration and dosage data) in the UK Biobank (Data Field: 20003) and classified these medications into 23 categories using the Anatomical Therapeutic Chemical (ATC) Classification System, a system developed by the WHO that classifies all drugs into definite groups based on their therapeutic, pharmacological, and chemical properties [19]. Then, they performed 23 medication-use GWASs. The ATC code and corresponding drug category name are shown in Figure 1. The detailed drug list included in each ATC code and the particulars (such as case/control number) of 23 medication-use GWASs can be found in Santos R et al.’s study [19] and Wu et al.’s study [12], respectively. The 23 categories of medications represent common existing drugs in the clinic and link with specific mechanisms and drug targets, which have important clinical significance [12]. The medication-use GWAS data were also applied in recent MR studies; for example, evaluating the effect of opioid use on major depression [15,16,17].

### 2.2. Outcome Data: GWAS Summary Statistics for Three Major Neurodegenerative Diseases

We used summary statistics from the publicly available GWAS summary statistics for AD (21,982 cases and 41,944 controls) [20], PD (33,674 cases and 449,056 controls) [21] and ALS (20,806 cases and 59,804 controls) [22] of European ancestry (Figure 1). Via the R package ‘TwoSampleMR’ version 0.5.6 in R software version 4.0.3 (R Foundation for Statistical Computing, Vienna, Austria), we accessed the GWAS summary statistics for AD (GWAS ID: ieu-b-2), PD (GWAS ID: ieu-b-7) and ALS (GWAS ID: ebi-a-GCST005647) through the IEU OpenGWAS database API https://gwas.mrcieu.ac.uk/ (accessed on 23 October 2022).

### 2.3. MR Analysis

We assessed the relationships between 23 medication categories and three major neurodegenerative diseases (AD, PD and ALS) using a two-sample MR approach, in which the selections of IVs are based on GWAS summary statistics. Independent SNPs identified in each GWAS that reached the threshold (*p*-value < 5 × 10^−8^ and r^2^ < 0.001) were selected as instrumental variants (IVs) for each medication category. SNPs absent in the outcome data were replaced by proxy SNPs (LD r^2^ values for proxies at 0.8 and a MAF threshold for aligning palindromes at 0.3) [23]. The statistical power was calculated using the F-statistic.

We used the inverse variance-weighted (IVW) method as the principal MR analytical method to provide an overall estimate of the causal effect [24]. Sensitivity analysis, including weighted median [25] and MR-Egger regression methods [26], was additionally performed. Pleiotropy was evaluated by the MR-Egger intercept test [26]. If the MR-Egger intercept test showed pleiotropy (*p* < 0.05), meaning IV violations, we used the MR pleiotropy residual sum and outlier (MR-PRESSO) test [27] to identify the outliers driving the pleiotropy, and then excluded these outliers for performing a corrected MR analysis. To assess the robustness of the significant results, we also performed leave-one-out analyses to detect high influence points [28]. The results are expressed as odds ratios (ORs) and 95% confidence intervals (CIs) for per unit increase in log odds of the medication use. A multiple-testing adjusted *p* < 0.00072 (0.05 divided by 69 (23 × 3)), known as the Bonferroni correction, was defined as statistical significance. However, a *p* value above 0.00072 but below 0.05 was also considered evidence of a potential association. We performed all analyses using the ‘TwoSampleMR’ package version 0.5.6 in R software version 4.0.3 (R Foundation for Statistical Computing, Vienna, Austria. URL http://www.R-project.org/).

### 2.4. Further Analysis for Identified Associations between Medications and NDs

Medications are prescribed when primary diseases exist, so the genetic variants used as instruments are also associated with primary diseases. It is necessary to interpret whether the associations between medications and NDs are attributed to the primary diseases. If we find the potential associations between medications and NDs (*p* < 0.05), there will be three relationship possibilities (Figure S1 in Supplementary File S2): (1) the primary disease is associated with NDs independent of medication; (2) the association between primary disease is mediated by medication use; and (3) the medication is associated with NDs independent of primary disease, for example, through side effects. To further interpret the relationship between primary disease, medications and NDs, we first assess the associations between primary disease and NDs. The major primary disease that each medication category is prescribed for has been indicated in the previous work [12]. GWAS information about primary diseases is shown in Supplementary File S3. If there is no association between primary disease and NDs, it suggests the possibility that medication is associated with NDs independent of the primary disease. If the primary disease was associated with NDs, we compared the associations before and after removing instrumental variants associated with medications (*p*-value < 5 × 10^−8^) to indicate whether medication use mediates the associations.

## 3. Results

**Figure 2:**
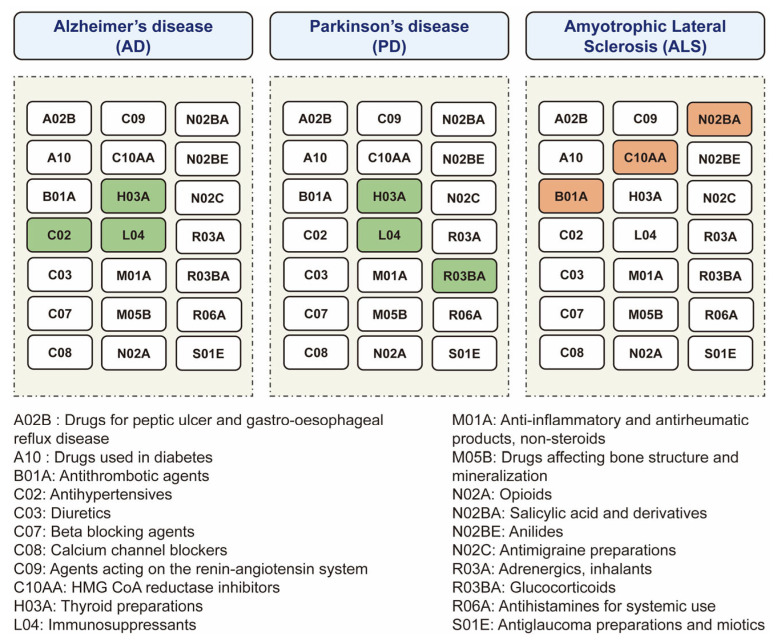
The overview diagram for the relationships between medications and neurodegenerative diseases. The significant associations between medications and diseases are shown. The color of each medication square represents the positive associations (red), negative associations (green), or no associations (white) with disease risk.

**Figure 3:**
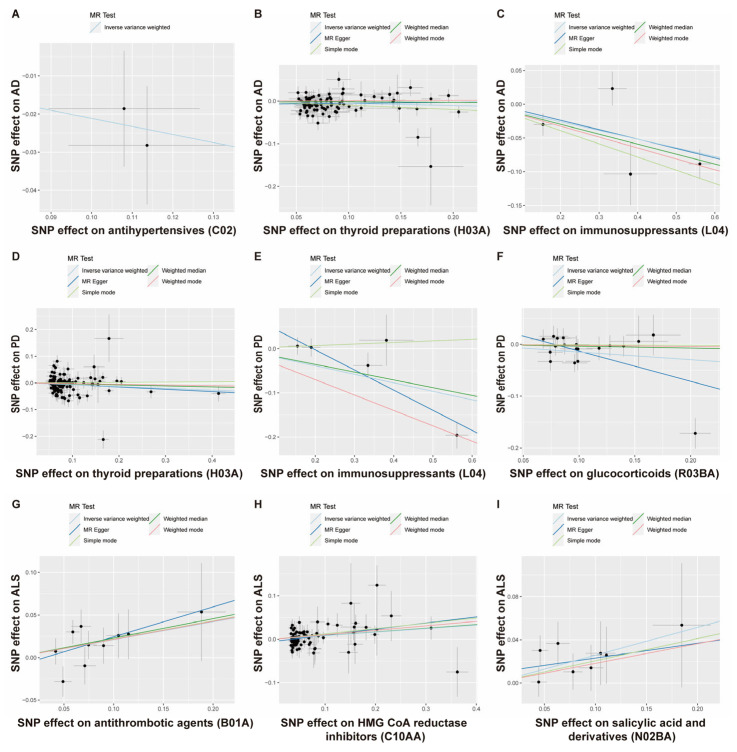
Scatterplot analysis. Scatterplot of instrument SNP effects of medication use on Alzheimer’s disease (**A**–**C**), Parkinson’s disease (**D**–**F**), and amyotrophic lateral sclerosis (**G**–**I**). Colored lines are the regression slopes fitted by the primary IVW MR method and other complementary methods.

Using the MR approach, we systematically screened the causal associations between 23 categories of existing medications and the risk of major neurodegenerative diseases, including AD, PD, and ALS (Figure 1). Because the vasodilators used in cardiac diseases (C01D) and antidepressants (N06A) only have one SNP achieving the *p*-value level of 5 × 10^−8^, the remaining 21 categories of medications were used to perform MR (Figure 2). All the F-statistics were above 10 (Supplementary File S1), indicating that there was no obvious weak instrument bias. After screening, we identified that a genetic predisposition to antihypertensives (C02), thyroid preparations (H03A), and immunosuppressants (L04) was causally associated with a decreased risk of AD, a predisposition to thyroid preparations (H03A), immunosuppressants (L04), and glucocorticoids (R03BA) was causally associated with a lower risk of PD, and a predisposition to antithrombotic agents (B01A), HMG CoA reductase inhibitors (C10AA), and salicylic acid and derivatives (N02BA) was causally associated with a higher risk of ALS (Figure 2 and Figure 3). In this way, we presented the whole picture with regard to the causal association between existing medications and major neurodegenerative diseases. The details of the analysis will be shown as follows.

### 3.1. The Putative Causal Relationship between Medication-Taking Traits and AD

**Figure 4:**
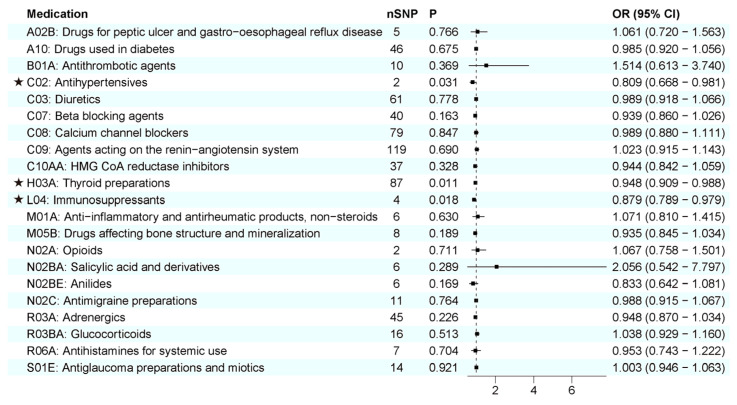
IVW results between 21 medication-taking traits and Alzheimer’s disease (AD). The number of SNP (nSNP) as instruments, odds ratio (OR), and 95% confidence interval (CI) are shown. Horizontal lines represent 95% CI and the square represents OR. The asterisk represents the presence of potential associations between medications and Alzheimer’s disease.

First, we examined the relationship between 21 genetically determined medication-taking traits and the risk of AD. The number of SNPs (IVs) for each medication and the IVW results are shown in Figure 4. Initially, IVW suggested four genetically determined medication-taking traits were associated with AD, including antihypertensives (nSNP = 2, OR = 0.809, 95% CI = 0.668–0.981, *p* = 0.031), thyroid preparations (nSNP = 93, OR = 0.960, 95% CI = 0.924–0.998, *p* = 0.040), immunosuppressants (nSNP = 4, OR = 0.879, 95% CI = 0.789–0.979, *p* = 0.018), and HMG CoA reductase inhibitors (nSNP = 65, OR = 1.269, 95% CI = 1.028–1.567, *p* = 0.027). However, the MR-Egger intercept analysis of thyroid preparations and HMG CoA reductase inhibitors showed horizontal pleiotropy (thyroid preparations: intercept, −0.010 ± 0.004, *p* = 0.019; HMG CoA reductase inhibitors: intercept, −0.043 ± 0.012, *p* = 0.001). Then, we used the method of MR-PRESSO to identify the outliers driving the horizontal pleiotropy and excluded these outliers for performing a new MR analysis. After excluding outliers, we repeated the MR-Egger intercept analysis, and no horizontal pleiotropy was indicated (thyroid preparations: intercept, −0.008 ± 0.005, *p* = 0.134; HMG CoA reductase inhibitors: intercept, −0.009 ± 0.008, *p* = 0.269). A total of 87 SNPs remained for thyroid preparations, and the IVW analysis showed a more significant association (OR = 0.948, 95% CI = 0.909–0.988, *p* = 0.011). In comparison, 65 SNPs remained for HMG CoA reductase inhibitors, and the IVW analysis showed no association with AD (*p* = 0.328). The IVW results were generally consistent with associations from the weighted median and MR-Egger methods (Figure 3A–C; Supplementary File S1). The leave-one-out analysis did not identify any single genetic variant with a high influence (Supplementary File S2). There was no association between the other 18 medication categories and AD, with *p* values > 0.05 in all of the analyses (Figure 4). Thus, these data indicated that genetic predisposition to antihypertensives, thyroid preparations, and immunosuppressants was associated with a decreased risk of AD. To further interpret whether the associations between medications (antihypertensives, thyroid preparations, immunosuppressants) and AD are attributed to the primary diseases, we assessed the effects of systolic blood pressure (SBP), hypothyroidism, and rheumatoid arthritis (RA) on AD, respectively. As shown in Table 1 and Supplementary File S3, there was no evidence for an association between genetically estimated SBP or hypothyroidism and AD, which indicates that SBP and hypothyroidism were not driving the associations between medications and AD. RA is negatively associated with AD (OR = 0.958, 95% CI = 0.922–0.996, *p* = 0.032). However, after removing the IVs associated with immunosuppressants, the association between RA and AD was absent (OR = 0.968, 95% CI = 0.923–1.015, *p* = 0.173), which suggests that the use of immunosuppressants may mediate the association between RA and AD.

### 3.2. The Putative Causal Relationship between Medication-Taking Traits and PD

**Figure 5:**
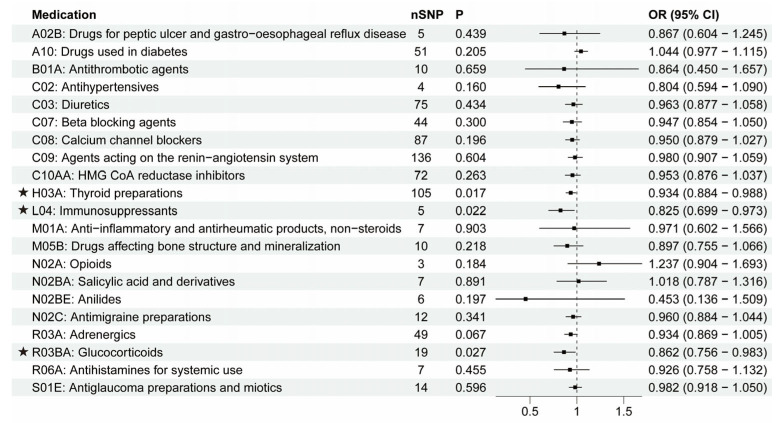
IVW results between 21 medication-taking traits and Parkinson’s disease (PD). The number of SNP (nSNP) as instruments, odds ratio (OR), and 95% confidence interval (CI) are shown. Horizontal lines represent 95% CI and the square represents OR. The asterisk represents the presence of potential associations between medications and Parkinson’s disease.

Next, the causal relationship between medication-taking traits and PD was also assessed by MR analysis. The number of SNP proxies for each medication and the IVW results are shown in Figure 5. It was suggested that genetically determined thyroid preparations (OR = 0.934, 95% CI = 0.884–0.988, *p* = 0.017), immunosuppressants (OR = 0.825, 95% CI = 0.699–0.973, *p* = 0.022), and glucocorticoids (OR = 0.862, 95% CI = 0.756–0.983, *p* = 0.027) were causally associated with a lower risk of PD. The associations were broadly consistent with associations from the sensitivity analyses, including weighted median and MR-Egger methods (Figure 3D–F; Supplementary File S1). The MR-Egger intercept analysis did not indicate horizontal pleiotropy (thyroid preparations: intercept, 0.002 ± 0.006, *p* = 0.758; immunosuppressants: intercept, 0.089 ± 0.032, *p* = 0.070; glucocorticoids: intercept, 0.045 ± 0.023, *p* = 0.066). We did not find a single genetic variant that drove the observed effect in the leave-one-out analysis (Supplementary File S2). The other 18 categories of medications showed no causal association with PD risk (Figure 5). Our results identified that a genetic predisposition to thyroid preparations, immunosuppressants, and glucocorticoids was causally associated with a lower risk of PD development. Then, we evaluated the effects of hypothyroidism, RA, and asthma on PD, respectively (Table 1; Supplementary File S3). We found no associations between hypothyroidism or asthma and PD, indicating that the associations between thyroid preparations/glucocorticoids and PD were not very likely to be attributed to the primary diseases. At the same time, RA was negatively associated with PD (OR = 0.926, 95% CI = 0.886–0.969, *p* = 0.001). However, after removing the IVs associated with immunosuppressants, the association between RA and PD was absent (OR = 0.947, 95% CI = 0.893–1.005, *p* = 0.071), suggesting the possibility that immunosuppressants mediated the association between RA and PD.

### 3.3. The Putative Causal Relationship between Medication-Taking Traits and ALS

**Figure 6:**
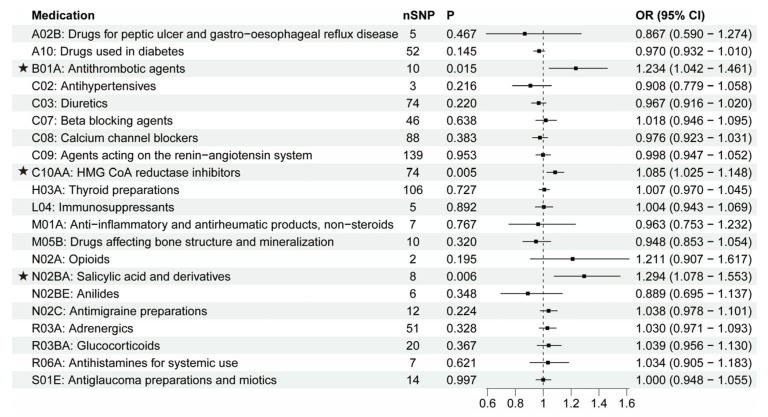
IVW results between 21 medication-taking traits and amyotrophic lateral sclerosis (ALS). The number of SNP (nSNP) as instruments, odds ratio (OR), and 95% confidence interval (CI) are shown. Horizontal lines represent 95% CI and the square represents OR. The asterisk represents the presence of potential associations between medications and ALS.

Finally, we used the MR approach to identify the causal relationship between 21 categories of medications and ALS. As shown in Figure 6, three medications were found to have suggested associations with higher ALS development risk: antithrombotic agents (OR = 1.234, 95% CI = 1.042–1.461, *p* = 0.015), HMG CoA reductase inhibitors (OR = 1.085, 95% CI = 1.025–1.148, *p* = 0.005), and salicylic acid and derivatives (OR = 1.294, 95% CI = 1.078–1.553, *p* = 0.006). IVW and other sensitivity analyses were generally consistent (Figure 3G–I; Supplementary File S1). The Egger analysis did not show directional pleiotropy (antithrombotic agents: intercept, −0.011 ± 0.020, *p* = 0.597; HMG CoA reductase inhibitors: intercept, −0.005 ± 0.003, *p* = 0.166; salicylic acid and derivatives: intercept, 0.009 ± 0.018, *p* = 0.621). There was no distortion in the leave-one-out plot, meaning that no single SNP was driving the observed effect (Supplementary File S2). The other 18 categories of medications showed no causal association with ALS risk (Figure 6). Our findings showed that antithrombotic agents, HMG CoA reductase inhibitors, salicylic acid and derivatives were positively causally associated with a higher ALS risk. B01A (antithrombotic agents) and N02BA (salicylic acid and derivatives) showed a similar pattern in associations with diseases because the two medication categories both include the original medication aspirin, which has multiple ATC codes (A01AD05, B01AC06 and N02BA01) [8], and coronary artery disease (CAD) is the major primary disease for aspirin. Thus, we then explored the causal effects of CAD and low density lipoprotein cholesterol (LDL-C) on ALS. In Table 1 and Supplementary File S3, it is shown that CAD had no causal association with ALS while LDL-C was positively associated with ALS (OR = 1.075, 95% CI = 1.013–1.141, *p* = 0.017). After removing the IVs associated with HMG CoA reductase inhibitors, the association between LDL-C and ALS was absent (OR = 1.036, 95% CI = 0.949–1.131, *p* = 0.432). These data indicated that the associations between B01A / N02BA and ALS were not due to the primary disease CAD, and the use of HMG CoA reductase inhibitors (statins) may contribute to the LDL-C-related ALS risk.

## 4. Discussion

Our MR study systematically screened for the causal relationships between existing medications and major NDs and exhibited a whole picture for these relationships. There were seven potential categories of medications causally associated with NDs: immunosuppressants, glucocorticoids, salicylic acid and derivatives, antithrombotic agents, HMG CoA reductase inhibitors, antihypertensives, and thyroid preparations. These results are supported by some of the scattered observational epidemiological literature and animal-based evidence [17,29,30,31,32,33,34,35,36,37]. Establishing these relationships not only deepens our understanding of the nature and mechanisms of neurodegenerative diseases, but also potentially opens the rapid green way for treatment and prevention.

From the methods aspect, there are already several kinds of genetic proxies used as IVs in MR to study drug effects. These include genetic proxies for the mechanisms targeted by these medications (e.g., LDL-lowering variants in the gene encoding HMGCR for statins), genetic proxies for the expression of specific genes that are drug targets, and genetic proxies for the response to these medications. However, these proxies are mostly derived by assessing specific markers in circulating blood rather than the central nervous system or specific neurons in situ [38,39,40]. The blood–brain barrier (BBB) tightly controls the movement of ions, molecules, and cells between the blood and the brain so that the central nervous system is relatively independent and has different patterns from other peripheral tissues in metabolism, immunity, etc. The BBB also influences drug delivery to the central nervous system [41]. Thus, for many drugs or drug targets, such as those used in or being developed for treating neurological conditions, a circulating biomarker may not represent a strong proxy for the drug target. Evidence shows that gene expression-based MR estimates may differ in magnitude and even direction across different tissues [38]. On the other hand, these proxies predict solely on-target effects of drug use; they do not encapsulate off-target consequences of related therapeutic medications. However, the ‘off target’ of existing medications is usually critical for drug repositioning. Our study employed a medication-use GWAS to extract IVs, which provides the possibility to evaluate the direct and total effect of existing medications on NDs (not identification of a single molecular target) and can screen the medications in categories systematically.

Because the specific category of medication targets a specific mechanism, our findings indicate several major mechanisms involved in the development of neurodegenerative diseases. Even though various NDs have the common feature that neurons progressively degrade, our data also give some clues that the major nature and mechanism of each ND are different from each other. First, we found that immunosuppressants (targeting immunity), antihypertensives (targeting blood pressure or vascular remodeling), and thyroid preparations (targeting thyroid function dysregulation) were negatively associated with AD, indicating that mechanisms involved in AD include immunity, vascular mechanism, and thyroid function dysregulation; second, our results showed that immunosuppressants, glucocorticoids (targeting immunity), and thyroid preparations (targeting thyroid function dysregulation) were negatively associated with PD, suggesting that mechanisms involved in PD include immunity and thyroid function dysregulation; third, our data showed that HMG CoA reductase inhibitors (targeting lipid metabolism dysfunction), antithrombotic agents, and salicylic acid and derivatives (mainly including aspirin and targeting inflammation or platelet function) were positively associated with risk of ALS, meaning that mechanisms involved in ALS may include lipid metabolism dysfunction, inflammation, or platelet dysfunction. These findings deepened the understanding of the specific nature of each ND, which provides the potential of precise targeting for specific ND types. For the quite confusing area of the NDs, which is full of varying and numerous hypotheses and theories, these causal associations between existing medications and NDs are worthy of more attention and a follow-up in the future. In addition to the above positive results, our work also showed meaningful noncausal associations between NDs and some medications. These drugs, such as opioids, drugs used in diabetes, were also previously found to be associated with the risk of neurodegenerative diseases, but it is not certain whether these associations are causal [42,43,44,45]. In our study, there was no association found between these medications and NDs. Reverse causation or comorbidities might be the main explanation for these associations. Therefore, our findings also helped to clarify and clear up the noncausal associations between medications and NDs.

Combining our work with previous findings will promote our understanding of the mechanisms by which medications influence NDs. If medication use is associated with NDs while the primary disease is not, we consider that the effect of medication on NDs may be attributed to an ‘off-target’ mechanism that is independent of the primary disease. For example, in our results, antihypertensives was associated with AD but SBP is not, so it is reasonable to conclude that antihypertensives can affect AD by some mechanisms (such as alleviating vascular remodeling or affecting ion channels) other than lowering blood pressure. Interestingly, a similar conclusion is also derived from previous work [46]. On the other hand, if primary disease and medication use are both associated with NDs with similar associations, there is the possibility that the medication prescribed for treating the primary disease is eventually the mediator to influence NDs rather than the primary disease itself. For example, our data showed rheumatoid arthritis and immunosuppressants are both associated with a lower risk of AD and PD; however, when we removed the IVs of rheumatoid arthritis associated with the use of immunosuppressants, the associations between rheumatoid arthritis and NDs were absent. Thus, it is possible that immunosuppressants contribute to a reduced risk of PD and AD in rheumatoid arthritis, which is also consistent with recent works [17,47].

Based on the understanding of the ND mechanisms and drug functional mechanisms, the framework of our MR studies will provide a potentially valuable resource to generate new leads relevant to optimizing drug repositioning, new drug target identification, and prediction of unfavorable side effects to promote precise medicine for NDs. (i) Drug repositioning. Our work is based on existing drugs in the clinic, some of which have potentially beneficial effects on specific neurodegenerative diseases. Thus, it will promote drug repositioning and provide an expressway for drug development because the profiles (such as safety and pharmacokinetics) of the repurposed drugs are already established. (ii) Patient management. Our findings will contribute to quantifying the future risk of medication taking for ND patients. Comorbidity is commonly observed in clinical practice. Our MR results highlight evidence that some existing medications might increase the risk of neurodegenerative diseases (risky medical exposures), thereby promoting precision medicine based on risk prediction and management. (iii) Guiding an aging population. Aging is a significant risk factor for many neurodegenerative diseases [48], and as individuals age, they may require multiple medications to manage various health conditions. Our research provides insights into identifying medications that may have a positive impact on the aging population and potentially modify the course of developing neurodegenerative diseases. This knowledge can guide healthcare professionals in prescribing appropriate medications and optimizing treatment strategies for elderly individuals, with the aim of improving their overall health outcomes and quality of life.

Our work also has limitations. In general, it is important to recognize the limitations of the MR approach. One of the key assumptions in MR is the validity of IVs. Violations of this assumption, such as pleiotropy, can introduce bias into the estimates. We acknowledge this limitation and emphasize the importance of careful selection of IVs and conducting sensitivity analyses to assess the robustness of the results. However, it is extremely difficult to completely rule out the horizontal pleiotropy and alternative direct causal pathway in MR tests. Another challenge in MR is the limited availability of appropriate IVs. It can be challenging to identify genetic variants that meet very precise criteria for IVs. This limitation can impact the precision and generalizability of the results. Specific limitations of our study are as follows: First, limited by the source data, the duration and dosage were not recorded. Thus, the dose and the duration of drug exposure differ among individuals and may change dynamically. Consequently, we could not identify SNPs associated with dosage levels, making accurate measurement of medication exposure very difficult. Second, the SNP associations were identified in only the European population aged 38–73 years, which may not be generalizable to other populations. Third, many *p*-values in our tests were below 0.05 but unfortunately did not survive in strict Bonferroni correction. However, we should caution against interpreting study findings solely based on a *p*-value threshold [49]. A *p*-value above the Bonferroni correction criteria but below 0.05 was also considered suggestive evidence for a potential association. Fourth, the medication–mechanism–disease axis is quite complex. Diseases caused by specific mechanisms will influence the behaviors of taking medications, and medications targeting specific mechanisms can also alleviate diseases to change the behaviors of patients. Moreover, the interactions have a spatial–temporal variation. There is no perfect approach to completely rule out the confounding caused by the intricate interactions between medications and diseases. Even RCTs cannot rule out this kind of confounding [50]. Thus, even though our study used genetically predicted data to reduce much confounding and reverse caution, and there are also many successful examples in detecting the causal associations between medications and diseases using medication-use GWAS [15,16,17,51], we still consider that it is quite difficult to clarify that the medications are beneficial or harmful only by this single study. It is more rigorous to combine different levels of evidence reported now or in the future and to consider the spatial–temporal variation.

## 5. Conclusions

Through an MR approach, our study systematically screened the potential causal associations between clinical medications and major NDs, indicating negative causal associations between immunosuppressants, antihypertensives, and thyroid preparations and AD risk, negative causal associations between immunosuppressants, glucocorticoids, and thyroid preparations and PD risk, and positive causal associations between HMG CoA reductase inhibitors, antithrombotic agents, and salicylic acid derivatives and ALS risk. This work provides us with a whole understanding of the relationship between medications and NDs. In addition to clarifying the nature and mechanisms of neurodegenerative diseases, this work also helps with drug repositioning and clinical management for ND patients.

## Figures and Tables

**Table 1:** IVW results between primary diseases and NDs before and after removing medication use associated SNPs.

Medication	Primary Disease	NDs	Associations between Primary Disease and ND (before Removing Medication Use Associated SNPs)			Associations between Primary Disease and ND (after Removing Medication Use Associated SNPs)		
			*p*	OR before	SNPs (N)	*p*	OR	SNPs (N)
Antihypertensives	Systolic blood pressure (SBP)	AD	0.399	0.997 (0.990–1.004)	--	--	--	--
Thyroid preparations	Hypothyroidism	AD	0.701	1.585 (0.150–16.706)	--	--	--	--
Immunosuppressants	Rheumatoid arthritis (RA)	AD	0.032	0.958 (0.922–0.996)	51	0.173	0.968 (0.923–1.015)	48
Thyroid preparations	Hypothyroidism	PD	0.172	0.088 (0.003–2.872)	--	--	--	--
Immunosuppressants	Rheumatoid arthritis (RA)	PD	0.001	0.926 (0.886–0.969)	50	0.071	0.947 (0.893–1.005)	47
Glucocorticoids	Asthma	PD	0.686	0.981 (0.894–1.077)	--	--	--	--
Antithrombotic agents/Salicylic acid and derivatives	Coronary artery disease (CAD)	ALS	0.285	1.034 (0.972–1.100)	--	--	--	--
HMG CoA reductase inhibitors	Low density lipoprotein cholesterol (LDL-C)	ALS	0.017	1.075 (1.013–1.141)	310	0.432	1.036 (0.949–1.131)	266

## Data Availability

Medication-use GWASs are available in Wu et al.’s study [12]. GWASs for different diseases can be assessed through the IEU OpenGWAS database API (https://gwas.mrcieu.ac.uk/). These have also been shown in Section 2.

## Supplementary Materials

The following supporting information can be downloaded at: https://www.mdpi.com/article/10.3390/biomedicines11071930/s1, Supplementary File S1: MR results; Supplementary File S2: Supplementary figures and codes; Supplementary File S3: Further analysis.
