# Supplementary material for: Systematic Screening of Associations between Medication Use and Risk of Neurodegenerative Diseases Using a Mendelian Randomization Approach
Source: Biomedicines. 2023 Jul 7;11(7):1930. doi: 10.3390/biomedicines11071930 (PMC10377701; doi:10.3390/biomedicines11071930)
Supplement: Supplementary file 1 [file biomedicines-11-01930-s001.zip › Supplementary File S2. Supplementary figures and codes.pdf]

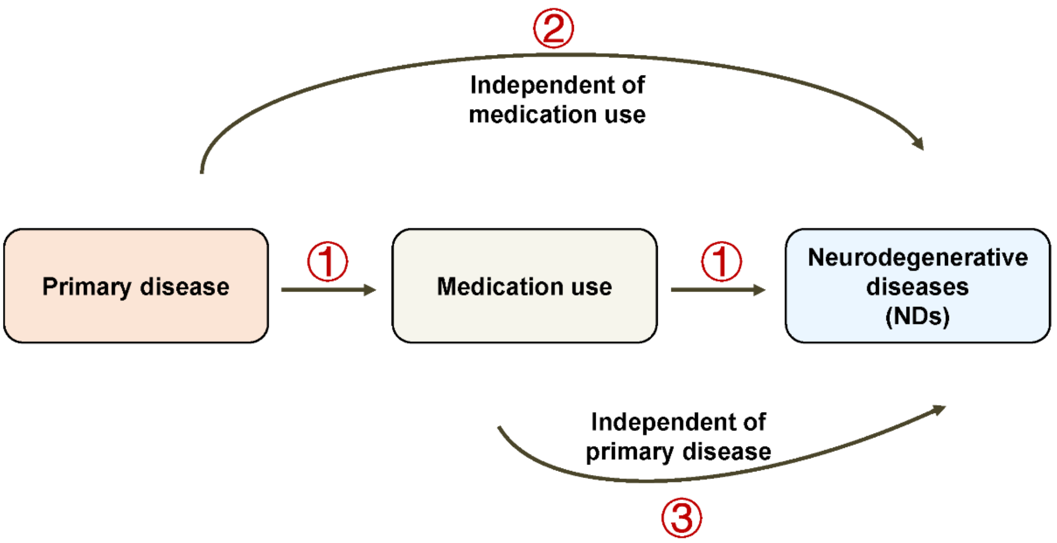

Figure S1. Three possibilities when medication use is associated with NDs: (1) the primary disease is associated with NDs independent of medication; (2) the association between primary disease is mediated by medication use; (3) the medication is associated with NDs independent of primary disease, for example, through side effects.

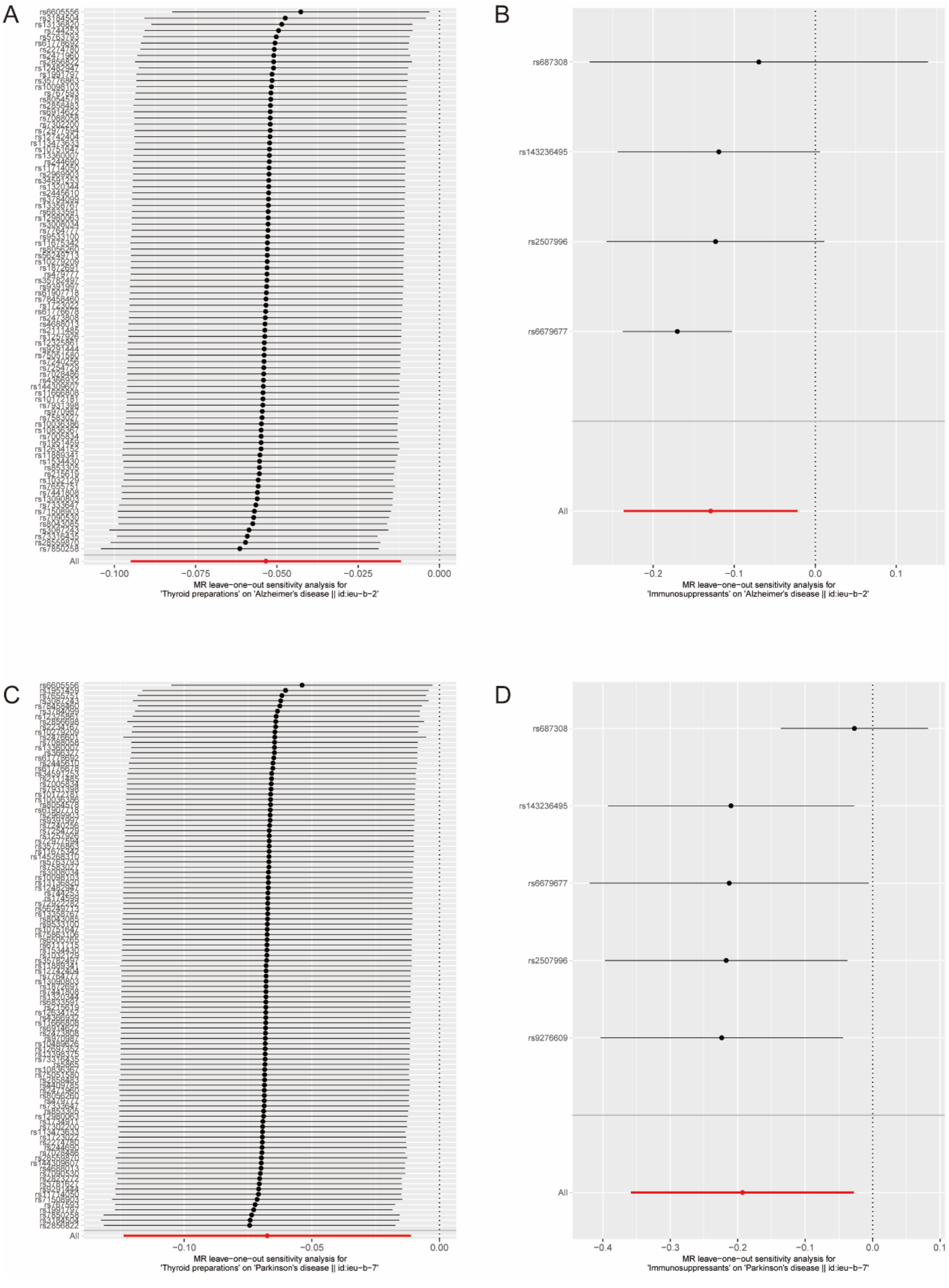
A
All
−0.100
−0.075
−0.050
−0.025
0.000
MR leave−one−out sensitivity analysis for
'Thyroid preparations' on 'Alzheimer's disease || id:ieu−b−2'
B
rs687308
rs143236495
rs2507996
rs6679677
All
−0.2
−0.1
0.0
0.1
MR leave−one−out sensitivity analysis for
'Immunosuppressants' on 'Alzheimer's disease || id:ieu−b−2'
C
All
−0.10
−0.05
0.00
MR leave−one−out sensitivity analysis for
'Thyroid preparations' on 'Parkinson's disease || id:ieu−b−7'
D
rs687308
rs143236495
rs6679677
rs2507996
rs9276609
All
−0.4
−0.3
−0.2
−0.1
0.0
0.1
MR leave−one−out sensitivity analysis for
'Immunosuppressants' on 'Parkinson's disease || id:ieu−b−7'

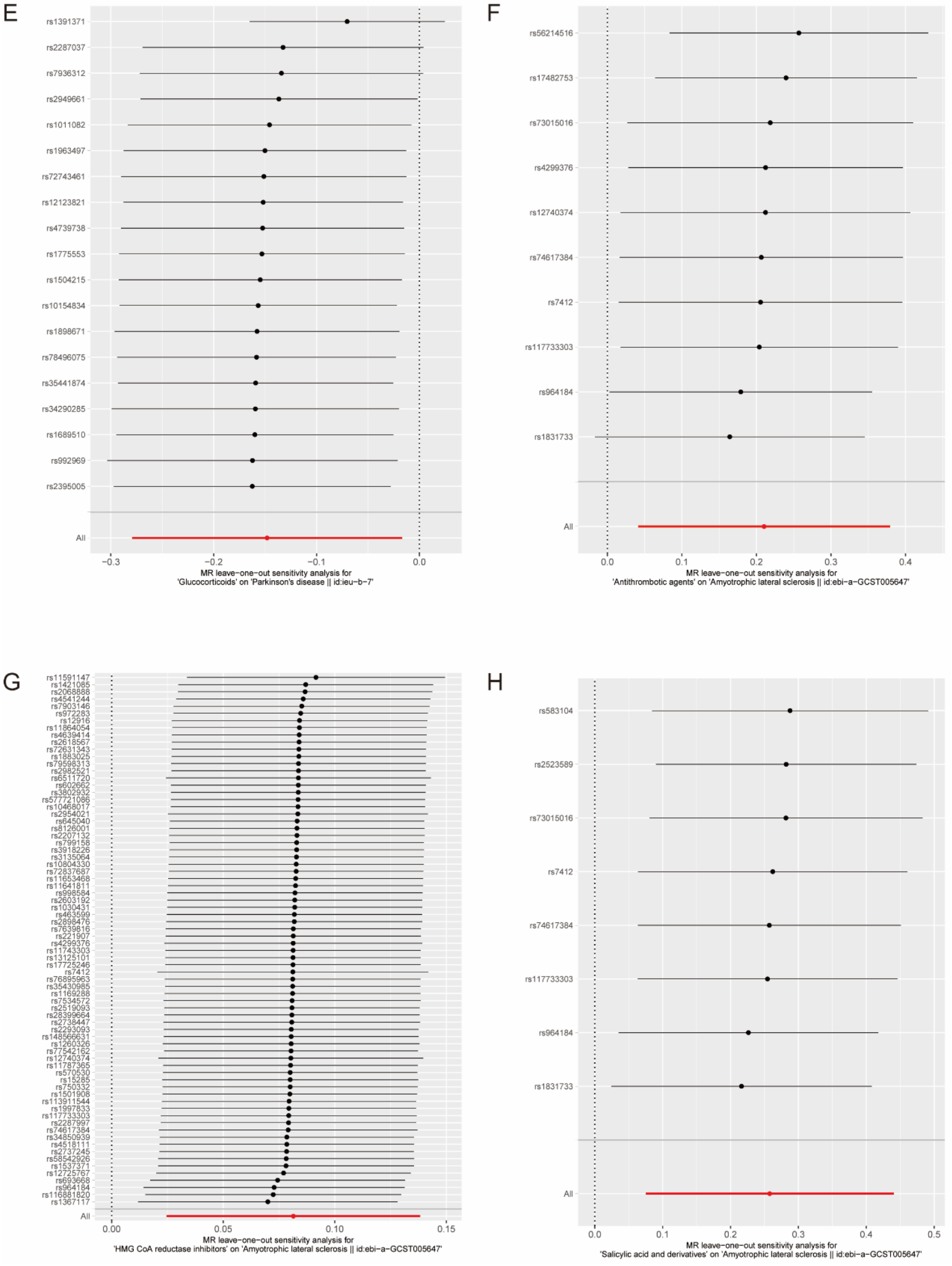

Figure S2. Forest plots of leave-one out sensitivity analysis between medications and neurodegenerative diseases. A-B shows the causal effect between thyroid preparations, immunosuppressants and Alzheimer's disease (AD); C-E shows the causal effect between thyroid preparations, immunosuppressants, glucocorticoids and Parkinson's disease (PD); F-H shows the causal effect between antithrombotic agents, HMG CoA reductase inhibitors, salicylic acid and derivatives and Amyotrophic Lateral Sclerosis (ALS).

#The codes for MR test on associations between medications and AD

```
library(TwoSampleMR)
library(MRInstruments)
setwd("C:\\Users\\Wenjing Wang\\Documents")
```

Taking the medication category C07 as an example:

```
##Obtain Genetic Instruments
C07_exp_dat <- read_exposure_data(
    filename = "C07_summary.csv",
    sep = ",",
    snp_col = "rsid",
    phenotype_col= "Traits",
    beta_col = "effect",
    se_col = "se",
    effect_allele_col = "a1",
    other_allele_col = "a2",
    eaf_col = "a1_freq",
    pval_col = "p-value",
    samplesize_col = "n"
)
head(C07_exp_dat)

C07_exp_dat <- clump_data(C07_exp_dat)
```

or

```
C07_exp_dat <- clump_data(
    C07_exp_dat,
    clump_kb = 10000,
    clump_r2 = 0.001,
    clump_p1 = 1,
    clump_p2 = 1,
    pop = "EUR"
)

## Extract Exposure IVs from Outcome GWAS and Harmonize
AD_out_dat <- extract_outcome_data(snps=C07_exp_dat$SNP,outcomes='ieu-b-2')
AD2_out_dat <- AD_out_dat[!duplicated(AD_out_dat$SNP), ]
dat <- harmonise_data(C07_exp_dat, AD2_out_dat)

## Perform MR Analysis
res <- mr(dat)
res
OR <- generate_odds_ratios(res)
OR
```

```
## Pleiotropy
pleiotropy <- mr_pleiotropy_test(dat)#Horizontal pleiotropy
pleiotropy

mr_presso(BetaOutcome = "beta.outcome", BetaExposure = "beta.exposure", SdOutcome = "se.outcome", SdExposure = "se.exposure", OUTLIERtest = TRUE, DISTORTIONtest = TRUE, data = dat, NbDistribution = 1000, SignifThreshold = 0.05)

## Scatter plot
p1 <- mr_scatter_plot(res, dat)
p1[[1]]

## Leaveoneout
res_loo <- mr_leaveoneout(dat)
p3 <- mr_leaveoneout_plot(res_loo)
p3[[1]]

## Forest plot
dt <- read.csv("ADMedicationForest.csv")
dt$"<- paste(rep(" ",20), collapse =" ")
dt$'OR (95% CI)' <- ifelse(is.na(dt$OR), "",
                           sprintf("%.3f (%.3f - %.3f)",
                                   dt$OR, dt$ORlci, dt$ORuci))
forest(dt[,c(1, 2, 3, 7, 8)],
       est = dt$OR,
       lower = dt$ORlci,
       upper = dt$ORuci,
       ci_column = 4,
       ref_line = 1)
```

#The codes for MR test on associations between medications and PD

```
library(TwoSampleMR)
library(MRInstruments)
setwd("C:\\Users\\Wenjing Wang\\Documents")
```

Taking the medication category C07 as an example:

```
##Obtain Genetic Instruments
C07_exp_dat <- read_exposure_data(
  filename = "C07_summary.csv",
  sep = ",",
  snp_col = "rsid",
  phenotype_col= "Traits",
```

```
  beta_col = "effect",
  se_col = "se",
  effect_allele_col = "a1",
  other_allele_col = "a2",
  eaf_col = "a1_freq",
  pval_col = "p-value",
  samplesize_col = "n"
)
head(C07_exp_dat)

C07_exp_dat <- clump_data(C07_exp_dat)
```

or

```
C07_exp_dat <- clump_data(
  C07_exp_dat,
  clump_kb = 10000,
  clump_r2 = 0.001,
  clump_p1 = 1,
  clump_p2 = 1,
  pop = "EUR"
)

## Extract Exposure IVs from Outcome GWAS and Harmonize
PD_out_dat <- extract_outcome_data(snps=C07_exp_dat$SNP,outcomes='ieu-b-7')
PD2_out_dat <- PD_out_dat[!duplicated(PD_out_dat$SNP), ]
dat <- harmonise_data(C07_exp_dat, PD2_out_dat)

## Perform MR Analysis
res <- mr(dat)
res
OR <- generate_odds_ratios(res)
OR

## Pleiotropy
pleiotropy <- mr_pleiotropy_test(dat)#Horizontal pleiotropy
pleiotropy

## Scatter plot
p1 <- mr_scatter_plot(res, dat)
p1[[1]]

## Leaveoneout
res_loo <- mr_leaveoneout(dat)
p3 <- mr_leaveoneout_plot(res_loo)
p3[[1]]
```

```
## Forest plot
dt <- read.csv("PDMedicationForest.csv")
dt$"<- paste(rep(" ",20), collapse =" ")
dt$'OR (95% CI)' <- ifelse(is.na(dt$OR), "",
                                             sprintf("%.3f (%.3f - %.3f)",
                                                     dt$OR, dt$ORlci, dt$ORuci))
forest(dt[,c(1, 2, 3, 7, 8)],
          est = dt$OR,
          lower = dt$ORlci,
          upper = dt$ORuci,
          ci_column = 4,
          ref_line = 1)
```

#The codes for MR test on associations between medications and ALS

```
library(TwoSampleMR)
library(MRInstruments)
setwd("C:\\Users\\Wenjing Wang\\Documents")
```

Taking the medication category C07 as an example:

```
##Obtain Genetic Instruments
C07_exp_dat <- read_exposure_data(
   filename = "C07_summary.csv",
   sep = ",",
   snp_col = "rsid",
   phenotype_col= "Traits",
   beta_col = "effect",
   se_col = "se",
   effect_allele_col = "a1",
   other_allele_col = "a2",
   eaf_col = "a1_freq",
   pval_col = "p-value",
   samplesize_col = "n"
)
head(C07_exp_dat)

C07_exp_dat <- clump_data(C07_exp_dat)
```

or

```
C07_exp_dat <- clump_data(
   C07_exp_dat,
   clump_kb = 10000,
   clump_r2 = 0.001,
```

```
  clump_p1 = 1,
  clump_p2 = 1,
  pop = "EUR"
)

## Extract Exposure IVs from Outcome GWAS and Harmonize
ALS_out_dat <- extract_outcome_data(snps=C07_exp_dat$SNP,outcomes=' ebi-a-GCST005647')
ALS2_out_dat <- ALS_out_dat[!duplicated(ALS_out_dat$SNP), ]
dat <- harmonise_data(C07_exp_dat, ALS2_out_dat)

## Perform MR Analysis
res <- mr(dat)
res
OR <- generate_odds_ratios(res)
OR

## Pleiotropy
pleiotropy <- mr_pleiotropy_test(dat)#Horizontal pleiotropy
pleiotropy

## Scatter plot
p1 <- mr_scatter_plot(res, dat)
p1[[1]]

## Leaveoneout
res_loo <- mr_leaveoneout(dat)
p3 <- mr_leaveoneout_plot(res_loo)
p3[[1]]

## Forest plot
dt <- read.csv("ALSMedicationForest.csv")
dt$''<- paste(rep(" ",20), collapse =" ")
dt$'OR (95% CI)' <- ifelse(is.na(dt$OR), "",
                           sprintf("%.3f (%.3f - %.3f)",
                                   dt$OR, dt$ORlci, dt$ORuci))
forest(dt[,c(1, 2, 3, 7, 8)],
       est = dt$OR,
       lower = dt$ORlci,
       upper = dt$ORuci,
       ci_column = 4,
       ref_line = 1)
```
